# Prevalence of heavy episodic drinking in the Brazilian adult population: National Health Survey 2013 and 2019

**DOI:** 10.1590/SS2237-9622202200003.especial

**Published:** 2022-06-29

**Authors:** Luiza Eunice Sá da Silva, Bruno Helman, Danilo Campos da Luz e Silva, Érika Carvalho de Aquino, Paula Carvalho de Freitas, Roberta de Oliveira Santos, Valéria Cristina de Albuquerque Brito, Leila Posenato Garcia, Luciana Monteiro Vasconcelos Sardinha

**Affiliations:** 1 Ministério da Saúde, Secretaria de Vigilância em Saúde, Brasília, DF, Brazil; 2 Instituto de Pesquisa Econômica Aplicada, Brasília, DF, Brazil

**Keywords:** Alcohol Drinking, Risk Factors, Health Surveys, Epidemiology Descriptive

## Abstract

**Objective:**

To describe the prevalence of heavy episodic drinking in the Brazilian adult population, according to sociodemographic characteristics, in 2013 and in 2019.

**Methods:**

A cross-sectional study using data on heavy episodic drinking among adults (≥ 18 years) from the National Health Survey, analyzed descriptively.

**Results:**

60,202 participants were included in 2013 and 88,531 in 2019. The prevalence of heavy episodic drinking, in 2019 (17.1%; 95%CI 16.6;17.5), was higher than 2013 (13.1%; 95%CI 13.1;14.2). In the two years, there was a higher prevalence among male sex, adults 18 to 39 years old, individuals with high schooling and Black skin color. In addition, higher prevalence were found among residents in urban areas and in the Midwest and Southeast regions.

**Conclusion:**

The indication of the growing prevalence of alcohol abuse in Brazil and the differences in prevalence, according to sociodemographic characteristics, show the need to encourage public policies and actions to combat its use.

**Study contributions**

**Main results**

An increase in the prevalence of alcohol abuse in the Brazilian population was identified, from 13.1% in 2013 to 17.1% in 2019, in addition to differences according to sociodemographic characteristics.

**Implications for the services**

The results of the study contribute to the promotion of public policies and their strengthening, in addition to actions to combat this important risk factor for disease burden and mortality in the Brazilian scenario.

**Perspectives**

Strengthening the perspective of comprehensive healthcare and reinforcing the intersectoral strategies, involving healthcare and regulatory bodies.

## Introduction

Alcohol is a psychoactive substance, lawfully used, widely accepted in the social environment, but whose consumption contributes to harm individuals and the community, in addition to posing economic burden on the healthcare system.^1^ Its use is an important risk factor for the development of diseases and for mortality across the globe.^1^ ^,^ ^2^

The World Health Organization (WHO) estimated that more than 40% of the population aged 15 or over, worldwide, consumed alcoholic beverages in 2016, which corresponded to over 2 billion people.^1^ The volume and patterns of alcoholic beverages intake, which vary according to sex, age group, socioeconomic status and effectiveness of public policies, have an impact on the population consumption and on health related consequences.^1^ ^-^ ^3^

Advancing knowledge about the harmful impact of alcohol intake on the health of individuals and populations is imperative, given the evidence of association of the substance with mortality and the occurrence of a wide variety of chronic diseases (cancers, cardiovascular diseases, liver diseases), some communicable diseases (HIV, tuberculosis and pneumonia), as well as accidents and violence.^1^ ^-^ ^3^ Alcohol consumption was listed as a cause associated with more than 200 diseases and conditions and a necessary causal factor in 40, according to the International Classification of Diseases.^1^

The concepts binge drinking and heavy episodic drinking were created based on evidence that, above a certain amount, the individual is at greater risk of having adverse effects related to its intake.^1^ However, the definition for what constitutes excessive drinking, the delimitation of a standard drink and alcohol percentage varies widely between countries and even within countries.

From 2013 to 2019, in Brazil alone, alcohol remained the seventh most relevant risk factor for mortality and the sixth most important for Disability-Adjusted Life Years (DALY), accounting for 5.5% of all deaths (77 thousand), and 5.7% of the total DALYs (3.16 million).^4^ Considering the harm attributed to heavy episodic drinking, in 2011 Brazil launched the Strategic Action Plan to Tackle Non-communicable Diseases (NCDs).^5^ Among other goals, this Plan proposed the reduction of abusive consumption of alcoholic beverages, defined as the consumption equal to or greater than five drinks in a single occasion for men, and equal to or greater than four drinks for women, by 10% by 2022.^5^

The consumption of alcoholic beverages also involves considerable costs not only for the consumers but society as a whole.^6^ According to a systematic review, the economic burden of alcohol on society is substantial, accounting for 0.45% to 5.44% of the gross domestic produc.^6^ In order to reduce the burden caused by the harmful use of alcohol and to offer risk-reducing benefits to vulnerable populations, WHO has identified actions that include: increasing taxes on alcoholic beverages; imposing restrictions on their display, advertising and physical availability in environments, in addition to drinking-driving legislation and enforcement measures.^1^ To that end, the Pan American Health Organization (PAHO) launched, in 2019, in Brazil, the SAFER initiative, which aims to promote actions at national and local levels to curb the consumption of alcohol, focused on interventions distributed in five strategic areas, supported by their cost-effectiveness and, consequently, their impact on public health.^7^

The available evidence is sufficient to place this matter as a priority on the agenda for public policies aimed at developing regulatory interventions to reduce the consumption of this product. Thus, monitoring alcohol consumption, especially through population surveys, can indicate the magnitude of the problem and contribute to the adoption of measures that collaborate to combat harmful alcohol consumption. Thus, the objective of this study was to describe the prevalence of heavy episodic drinking in the Brazilian adult population, according to sociodemographic characteristics, in 2013 and 2019.

## Methods

This was a cross-sectional descriptive study using data from the National Health Survey (PNS) carried out in 2013 and 2019. PNS is a population-based health survey, which collects representative data from the Brazilian household population, aiming to provide information on health conditioning factors, determinants and needs, being essential to guide actions and for the development of strategies and public policies in the area of healthcare.^8^

The survey sampling was carried out in three stages, by clusters. First, the stratification of the primary units (census tracts or groups of sectors) was performed, followed by simple random sampling of the households in the National Register of Addresses for Statistical Purposes (*Cadastro Nacional de Endereços para Fins Estatísticos*- CNEFE). Thirdly, for the interviews, a resident age ≥ 18 years old (2013) and ≥ 15 years old (2019) was randomly selected.^8^ The interviews were carried out between August 2013 and February 2014 (PNS 2013), and between August 2019 and March 2020 (PNS 2020), by means of mobile devices. More details concerning information about the PNS can be found in specific publication.^8^

For the present study, in order to maintain data comparability, only individuals age ≥ 18 years were included. Public data made available by the Instituto Brasileiro de Geografia e Estatística (IBGE) were used (https://bit.ly/3ySZ9cG, accessed November, 2020), without revealing the identity of the participants.

For the 2013 sample, the prevalence of heavy episodic drinking was calculated considering, as the numerator, the number of male participants who reported having consumed, on a single occasion in the 30 days prior to the interview, five or more alcoholic drinks and, in the case of females, four or more alcoholic drinks. The content of a drink was equivalent to about 12g of ethanol, that means about 60g for men and 48g for women. In 2019, however, the amount considered for heavy episodic drinking in the 30 days prior to the interview was five or more drinks for both males and females, fllowing WHO recommendations that characterize the abusive consumption of alcohol, in both sexes, by consumption of about 60g of alcohol on a single occasion within a period of 30 days.^1^ For this purpose, the following question was asked: *In the past 30 days, did you consume five or more alcoholic drinks on a single occasion?* A standard drink is defined as the equivalent to a shot of *cachaça* (sugar cane rum), a glass of wine, a can of beer, a shot of whiskey or any other distilled alcoholic beverage. Therefore, those individuals who responded affirmatively to this question were validated as having engaged in heavy episodic drinking, regardless of the number of occurrences. For prevalence estimates, the total number of individuals (≥ 18 years old) interviewed each year was used as the denominator.

The prevalence and their 95% confidence intervals (95%CI) were estimated for the Brazilian adult population, in 2013 and 2019, stratified according to: sex (male; female), age group (18 to 24; 25 to 39; 40 to 59; ≥ 60), level of education (no schooling and incomplete primary education; complete primary education and incomplete secondary education; complete secondary education and incomplete higher education; complete higher education); race/skin color (White; Black; Brown; Yellow; Indigenous), region (North; Northeast; Southeast; South; Midwest), and household location (urban; rural). For the Federative Units (UFs), prevalence and 95%CI were calculated according to sex and age groups.

The selected variables are described according to prevalence and 95%CI, considering the difference in the comparison of categories no interval overlap. All analyses were performed using a standard software package (Stata, version 14.2; StataCorp), through the survey module functions, in order to generate weighted estimates for the adult population in the country.

It is worth noting that PNS was approved by the National Research Ethics Committee/National Health Council (*Comissão Nacional de Ética em Pesquisa* - CONEP) under Opinion No. 3.529.376, issued on August 23, 2019. Informed consent was obtained at the time of the interview and signed on the data collection device.

## Results

In the 2013 PNS, collected interviews with 60,202 people aged 18 or over, corresponding to 48.3% male and 51.7% female. In the 2019 PNS, 88,531 people of the same age were interviewed, 46.8% male and 53.2% female.

The prevalence of abusive alcoholic beverages consumption in the 30 days prior to the interview, in 2019, was 17.1% (95%CI 16.6;17.5), showing an increase of 13.7% (95%CI 13.1;14.2) in relation to the results obtained in 2013.

Comparing the data from 2013 and 2019, the prevalence was higher in all categories of the sociodemographic variables studied in 2019. Higher proportions were found among males, being 21.6% (95%CI 20.7;22.5) in 2013, and 26.0% (95%CI 25.2;26.8) in 2019. Among females, it increased from 6.6% (95%CI 6.1;7.1) in 2013 to 9.2% (95%CI 8.7;9.7) in 2019. In the analysis of age groups, the highest prevalence was found among adults aged 18 to 24 (17.2%; 95%CI 15.7;18.8 in 2013, and 22.9%; 95%CI % 21.3;24.5 in 2019), and from 25 to 39 (18.9%; 95%CI 17.9;19.8 in 2013, and 23.7%; 95%CI 22.7;24, 7 in 2019), as shown in Table 1.

**Table 1:** Prevalence (%) of heavy episodic drinking, in the 30 days prior to the interview, among people age ≥ 18 years, according to sociodemographic characteristics, National Health Survey (PNS) 2013 (n = 60,202) and 2019 (n = 88,531), Brazil

Variables	% (95%CI^a^)	
	PNS 2013	PNS 2019
**Total**	13.7 (13.1;14.2)	17.1 (16.6;17.5)
**Sex**		
Male	21.6 (20.7;22.5)	26.0 (25.2;26.8)
Female	6.6 (6.1;7.1)	9.2 (8.7;9.7)
**Age group (years)**		
18 to 24	17.2 (15.7;18.8)	22.9 (21.3;24.5)
25 to 39	18.9 (17.9;19.8)	23.7 (22.7;24.7)
40 to 59	12.1 (11.4;12.8)	16.2 (15.4;17.0)
≥ 60	4.2 (3.6;4.8)	5.8 (5.3;6.2)
**Education**		
No schooling and incomplete primary education	11.1 (10.5;11.8)	12.7 (12.0;13.4)
Complete primary education and incomplete secondary education	15.8 (14.4;17.2)	20.6 (19.2;21.9)
Complete secondary education an incomplete higher education	15.4 (14.4;16.3)	19.2 (18.4;20.1)
Complete higher education	14.3 (12.8;15.8)	18.7 (17.5;19.9)
**Race/skin color**		
White	12.4 (11.7;13.2)	16.0 (15.3;16.8)
Black	16.6 (14.9;18.4)	19.6 (18.3;20.8)
Brown	11.2 (7.6;14.8)	17.5 (16.8;18.2)
Yellow	14.4 (13.7;15.1)	12.7 (9.4;17.1)
Indigenous	12.6 (7.7;17.5)	15.5 (11.4;20.7)
**Region**		
North	14.2 (12.9;15.4)	16.7 (15.8;17.7)
Northeast	15.6 (14.8;16.4)	17.3 (16.6;18.0)
Southeast	12.8 (11.9;13.7)	17.4 (16.5;18.2)
South	11.1 (10.0;12.2)	14.7 (13.8;15.6)
Midwest	16.2 (15.0;17.3)	19.6 (18.4;20.7)
**Household location**		
Urban	14.2 (13.6;14.8)	17.6 (17.1;18.1)
Rural	10.3 (9.2;11.3)	13.6 (12.7;14.5)

a) 95%CI: 95% confidence interval.

Regarding education, there was lower prevalence among people with no schooling or with incomplete primary education: 11.1% (95%CI 10.5;11.8) in 2013, and 12.7% (95%CI 12.0;13.4) in 2019. As for race/skin color, higher prevalence of heavy episodic drinking was found among people who reported Black race/skin color (16.6%; 95%CI 14.9;18.4 in 2013, and 19.6%; 95%CI 18.3;20.8 in 2019) compared to those who reported White race/skin color (12.4%; 95%CI 11.7;13.2 in 2013, and 16.0%; 95%CI 15.3;16.8 in 2019) (Table 1).

In 2019, the Midwest (19.6%; 95%CI 18.4;20.7) and Southeast (17.4%; 95%CI 16.5;18.2) regions had the highest prevalence, while the South region (14.7%; 95%CI 13.8;15.6) showed the lowest, as in 2013. Adults residing in urban areas reported higher rates of alcohol abuse (14.2%; 95%CI 13.6;14.8 in 2013, and 17.6%; 95%CI 17.1;18.1 in 2019), compared to residents in rural areas (Table 1).

**Figure 1:**
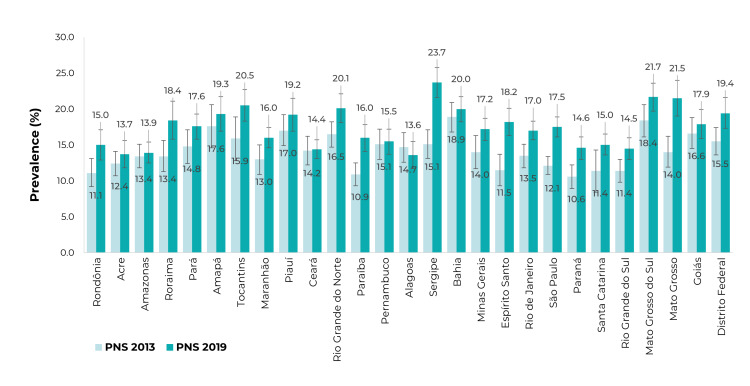
Prevalence (%) of heavy episodic drinking, in the 30 days prior to the interview, among people age ≥ 18 years, by Federative Units, National Health Survey (PNS) 2013 (n = 60,202) and 2019 (n = 88,531), Brazil

The increase in the prevalence of alcohol abuse was also observed in most Federative Units (UFs) (Figure 1). We highlight those with the highest prevalence in 2013 Bahia (18.9%; 95%CI 16.8;20.9), Mato Grosso do Sul (18.4%; 95%CI 16.1;20.6), and Amapá (17.6%; 95%CI 14.6;20.6). In 2019, the UFs with the highest prevalence were Sergipe (23.7%; 95%CI 21.6;25.8), Mato Grosso do Sul (21.7%; 95%CI 19.7;23.6) and Mato Grosso (21.5%; 95%CI 19.0;24.0).

**Figure 2:**
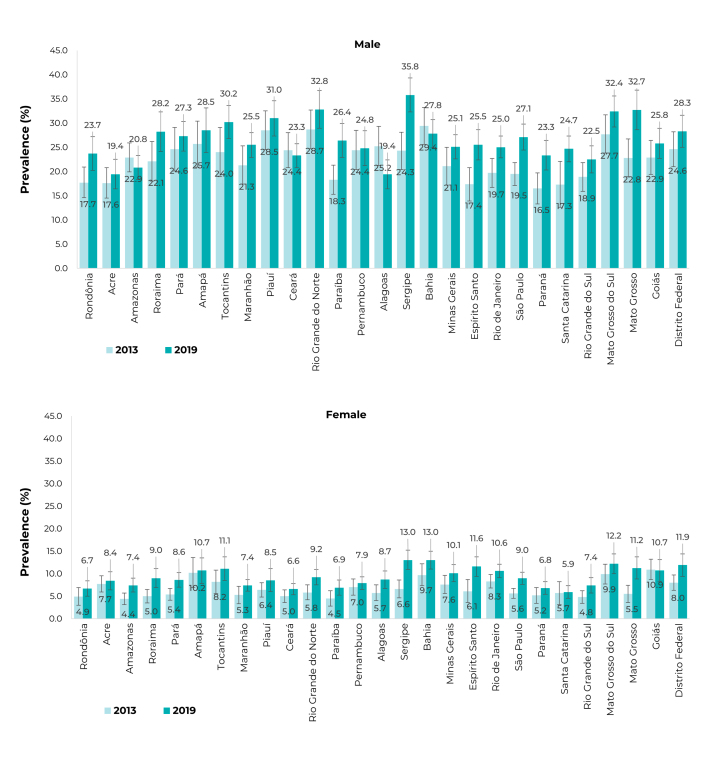
Prevalence (%) of heavy episodic drinking, in the 30 days prior to the interview, among people age ≥ 18 years, by Federative Units, according to sex, National Health Survey (PNS) 2013 (n = 60,202) and 2019 (n = 88,531), Brazil

In 2013, the highest prevalence among males were observed in Bahia (29.4%; 95%CI 25.6;33.2), Rio Grande do Norte (28.7%; 95%CI 24.7;32.7), and Piauí (28.5%; 95%CI 24.5;32.5); while in 2019, states such as Sergipe (35.8%; 95%CI 32.3;39.3), Rio Grande do Norte (32.8%; 95%CI 28.9;36.7), and Mato Grosso do Sul (32.7%; 95%CI 28.6;36.8) presented indicators above 30%. As for females, in 2013, the highest prevalence occurred in Goiás (10.9%; 95%CI 8.6;13.1), Amapá (10.2%; 95%CI 6.8;13.5), and Mato Grosso do Sul (9.9%; 95%CI 7.7;12.1); in 2019, Bahia (13.0%; 95%CI 11.0;15.0), Sergipe (13.0%; 95%CI 10.8;15.1), and Mato Grosso do Sul (12.2%; 95%CI% 10.0;14.4), as shown in Figure 2.

When stratified by UFs and age group, in 2019, the highest prevalence was observed in the 18 to 24 age group, mainly in Bahia (32.7%; 95%CI 25.0;40.4), Tocantins (30.3%; 95%CI 21.7;38.9), and Rio Grande do Sul (28.6%; 95%CI 22.3;35.0). For those aged 25 to 39, in Sergipe (32.9%; 95%CI 29.3;36.6), Mato Grosso (31.1%; 95%CI 26.4;35.9), and Mato Grosso do Sul (29.3%; 95%CI 26.0;32.7); between 40 and 59 years old, Sergipe (22.0%; 95%CI 19.0;25.0), Mato Grosso do Sul (20.5%; 95%CI 17.9;23.1), and Tocantins (19.3%; 95%CI 15.5;23.1); ≥ 60 years old: Amapá (9.0%; 95%CI 4.1;14.0), Rio Grande do Norte (8.8%; 95%CI 6.3;11.3), and Mato Grosso do Sul (8.5%; 95%CI 6.0;10.9) (Figure 3A; Figure 3B).

**Figure 3A:**
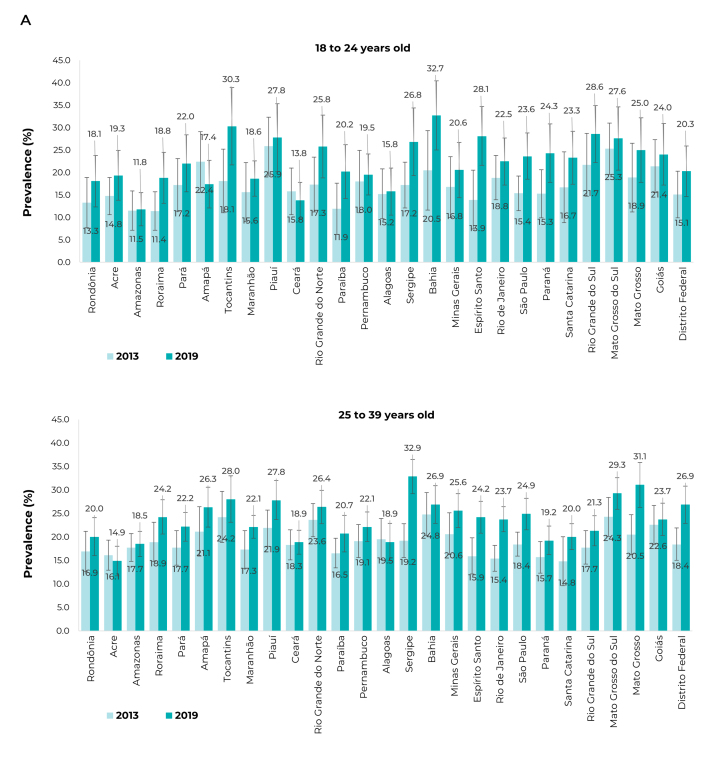
Prevalence (%) of heavy episodic drinking, in the 30 days prior to the interview, among people age ≥ 18 years, by Federative Units, according to age groups, National Health Survey (PNS) 2013 (n = 60,202) and 2019 (n = 88,531), Brazil

**Figure 3B:**
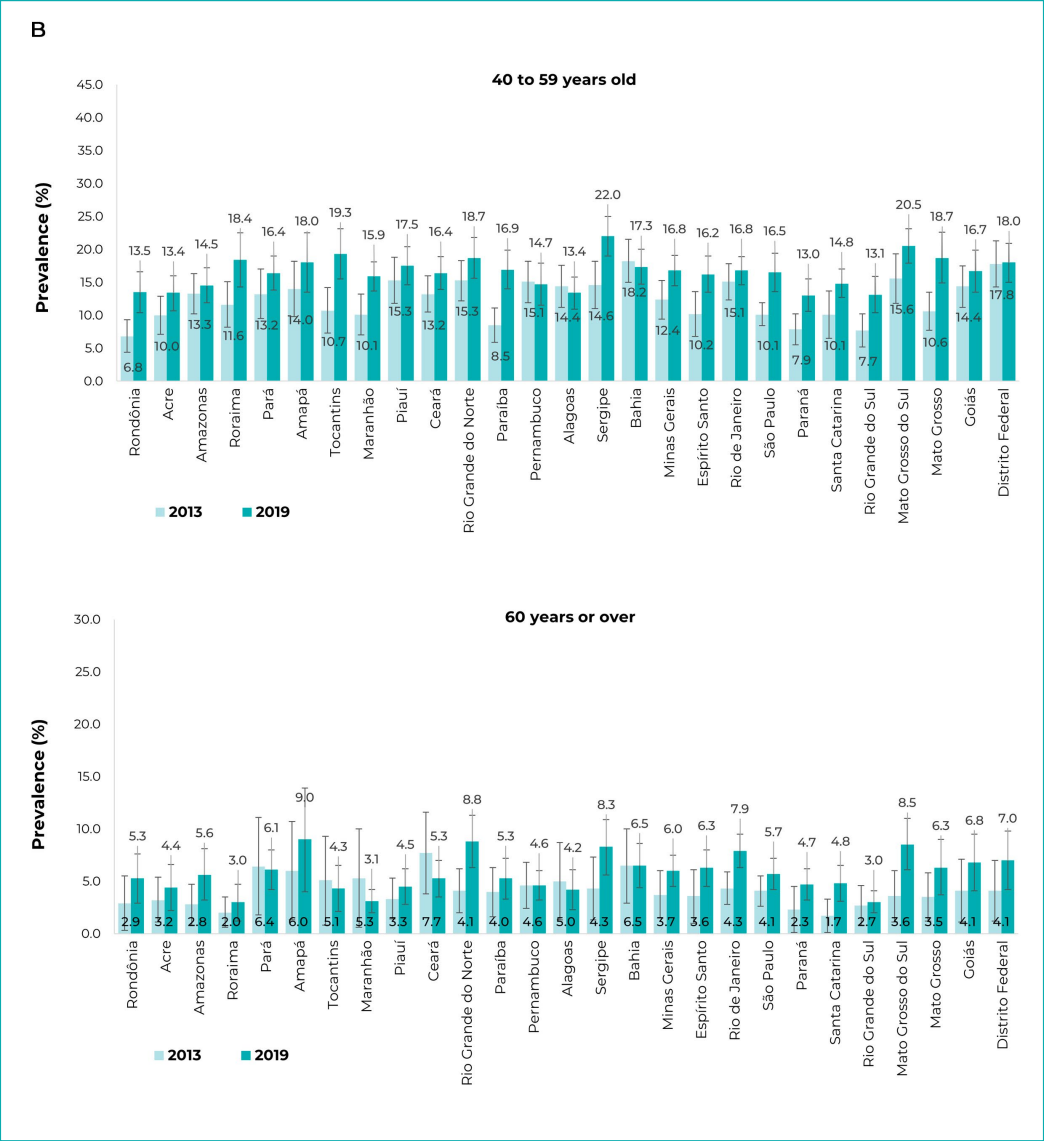
Prevalence (%) of heavy episodic drinking, in the 30 days prior to the interview, among people age ≥ 18 years, by Federative Units, according to age groups, National Health Survey (PNS) 2013 (n = 60,202) and 2019 (n = 88,531), Brazil

## Discussion

In 2019 the prevalence of heavy episodic drinking in the adult Brazilian population was higher than what was observed for 2013 for the country as a whole and most of the UFs. The increase in prevalence was also observed in the categories of sociodemographic characteristics studied. In the two years of the survey, higher prevalence was observed among males, young adults, people residing in urban areas and in the Midwest and Southeast regions, and with Black skin color. Lower prevalence was observed for individuals with no schooling or incomplete primary education when compared to individuals in the other educational strata.

The present study revealed findings consistent with previous publications that showed that male individuals have higher alcohol consumption than females. ^9^ ^,^ ^10^ However, despite this higher prevalence of males in 2019, there was an even greater increase in the period in females, around 39%. It is worth mentioning that, in 2013, the consumption of 4 or more doses of alcoholic beverages was considered as abusive consumption for females, and in 2019 this value increased to 5 or more doses. Thus, even with the need for greater consumption to be classified as abusive, there was an increase in the prevalence in the period for females.

Analysis of the temporal evolution of heavy episodic drinking among adults residing in Brazilian capitals indicated, from 2006 to 2013, that the consumption tendency remained stable for the whole population and for both sexes.^11^ However, between 2006 and 2019, an increase was observed for both the total adult population and females, with no similar trend being shown for the male population.^12^

A qualitative study conducted by Rodríguez, Moreno and Gómez (2019),^13^ relating gender and alcohol consumption, suggested that the inclusion of alcoholic beverage consumption into the female routine in the past decades has been tied to women’s incorporation into leisure spaces and practices that have traditionally been considered male. In addition, over the past years, women have been experiencing higher exposure to marketing involving alcoholic beverages, with a lot of advertisement focusing on sweeter alcoholic beverages and sparkling beverages, aimed specifically at the female consumer.^14^

Assessing the occurrence of a higher prevalence of abusive alcohol ingestion among younger age groups is also coherent with other national and international studies.^9^ ^,^ ^12^ ^,^ ^15^ According to data from the National Epidemiologic Survey on Alcohol and Related Conditions (NESARC), carried out in 2001-2002 with the United States’ adult population, over three quarters of the young adults aged between 21 and 24 years were current drinkers, and almost two thirds of those aged 18 to 20 years.^15^ Research of a qualitative nature has been carried out in an attempt to understand the meaning of alcohol abuse among young people and several studies suggest that this behavior is linked to the idea that abusive alcohol consumption is characteristic of this stage of life and normative for this period, intimately related to the process of reaching maturity.^16^ ^,^ ^17^ Furthermore, there is also the existence of intensive marketing strategies aimed at this public, with heavy emphasis on unregulated advertising for beer and easy access to alcohol in bars, parties and stores, justified by its high social acceptance in Brazil.^18^

On the other hand, significant increase of alcohol consumption among older individual is also in line with national^9^ ^,^ ^12^ and international studies.^14^ Among the reasons for such finding there is the use of alcohol related to low social involvement, illness, grief, changes in the routine, and economic problems.^19^

In both 2013 and 2019, abusive alcoholic beverage consumption was higher in the lower educational level strata. An increased prevalence was observed in all categories analyzed, even though they were unequal: the highest increment occurred in the population that reported complete higher education, with an increase of 30.8%; the lowest prevalence, on the other hand, was observed among those with no schooling or incomplete primary education. This finding can be explained by less access to alcoholic beverages due to the lower purchasing power of individuals in this educational stratum.^20^

For the other strata, it was found that abusive alcohol consumption was inversely proportional to the educational level, that is, the more years of education, the lower the prevalence of abusive use of alcohol. The 2019 PNS data also showed that the prevalence of alcohol consumption once or more times a week is directly proportional to education, that is, the more years of education, the higher the consumption.^21^ Even though this seems paradoxical, these findings can be explained by the fact that there is a higher risk of alcohol abuse and/or dependence among the population in the lower socioeconomic strata.^22^ ^,^ ^23^

Therefore, although alcohol consumption is less frequent among populations of lower socioeconomic status, they are more likely to be negatively affected by alcohol, leading to outcomes such as abusive consumption, dependence, the occurrence of diseases caused by alcohol and death.^22^ In this sense, the increase in prevalence of both regular consumption and abusive consumption, between 2013 and 2019, and the observation that the greatest growth occurred among the population with complete secondary education or incomplete higher education, can be explained by the effects of the global economic crisis, which affected the Brazilian population, yet differently according to the individual’s socioeconomic strata.^24^

A study that analyzed data from the 2013 PNS identified that individuals who reported Black and Brown race/skin color were associated with higher rates of heavy episodic drinking regardless of sex.^9^ Studies focusing specific populations - elderly, rural workers and urban workers - showed different results in this respect, with higher rates of heavy episodic drinking among individuals with white and brown skin color.^25^ ^-^ ^27^ The inconsistency of these results can be attributed to differences in the methods and measures for assessing the harmful use of alcohol, but also to the difficulties in using self-reported race/skin color as an ethnic-racial description in Brazil, given its impact on health conditions, notably, on the use of alcohol and other drugs.^26^ ^,^ ^27^

The pattern observed in 2019, with higher abusive alcohol consumption in the Midwest and Southeast regions, differs from the results found by Vigitel in 2019, which pointed to Salvador, Distrito Federal and Palmas as the capitals with the highest prevalence.^5^ The study carried out by Marques et al. (2019),^28^ on the other hand, identified “pockets” of high mortality rates from causes attributable to alcohol consumption in certain municipalities in the Northeast, Southeast and Midwest. Another study found that, between 2010 and 2012, the highest mortality rates from causes completely attributable to the use of alcohol occurred in the Northeast and Midwest.^29^

Considering that the consumption in each location can be influenced by regional and cultural determinants,^1^ ^,^ ^3^ ^,^ ^29^ consistent patterns might not be observed. Nevertheless, the increase observed for most of the UFs, for both sexes and age groups investigated, reinforce the significance of the problem of abusive alcohol consumption by the Brazilian population. Studies analyzing the evolution of alcohol consumption highlight the uneven tendency by location and population strata, mainly through increased consumption in groups that previously showed lower prevalences.^12^ However, these findings provide relevant information, suggesting that strategies and public policies aimed at reducing harmful alcohol consumption should advance in order to reach groups at greater risk of exposure.

As for the limitations of the study, it should be emphasized that determining abusive alcohol consumption based on the condition reported by the interviewee, without measuring the amount of alcohol that was ingested, in addition to the variation in alcoholic content in each type of alcoholic beverage, can generate lack of precision in the estimates. Notwithstanding, this type of self-reported information is widely used in health surveys, proving to be a useful indicator, and a reliable and valid approach to monitor this risk factor in the population. The change in values to measure the indicator among females in 2019 - from four or more drinks to five or more drinks -, also constitutes an important limitation of the study because it makes it difficult to compare the information obtained. Still, even though this difference possibly reduced those classified for the outcome, it was found that the behavior proved to have grown for the group.

Despite these limitations, it is noteworthy that the accuracy of the estimates presented in the study consider the sampling carried out by the PNS, which well represents the total adult population of the country, of the regions, the UFs, the Brazilian capitals, and the Federal District.

The high and growing prevalence of abusive alcoholic beverage consumption in Brazil, confirmed in the present study, highlights the need to encourage public policies and actions to address this significant risk factor for disease burden and mortality, with an alarming scenario in the Brazilian context. In the scope of the healthcare sector, strengthening the perspective of comprehensive healthcare is important, by reinforcing actions ranging from health promotion to improvement of the healthcare network, both through the qualification of screening and diagnosis of abusive consumption and suitable offer of psychosocial rehabilitation. In addition, the indications in the extensive literature on the subject corroborate that intersectoral actions, involving healthcare and regulatory bodies, can produce effective results. For this reason, we believe that the SAFER initiative can help decision makers in the adoption of strategies, protecting public health against external interests.

In this sense, and in light of the results of the present study, reinforcing strategies aimed at reducing the availability of alcoholic beverages through restrictions on sales locations and hours of sale, regulation of advertising in the mass media, and the adoption of taxation and regulation policies, is particularly recommended, needless to say, in combination with the reinforcement of other strategies currently in force.
